# Type 2 Diabetes Mellitus and Cardiovascular Disease: Genetic and Epigenetic Links

**DOI:** 10.3389/fendo.2018.00002

**Published:** 2018-01-17

**Authors:** Salvatore De Rosa, Biagio Arcidiacono, Eusebio Chiefari, Antonio Brunetti, Ciro Indolfi, Daniela P. Foti

**Affiliations:** ^1^Department of Medical and Surgical Sciences, Magna Græcia University of Catanzaro, Catanzaro, Italy; ^2^Department of Health Sciences, Magna Græcia University of Catanzaro, Catanzaro, Italy

**Keywords:** type 2 diabetes mellitus, cardiovascular disease, genetic polymorphisms, high-mobility group A1 variant, epigenetics

## Abstract

Type 2 diabetes mellitus (DM) is a common metabolic disorder predisposing to diabetic cardiomyopathy and atherosclerotic cardiovascular disease (CVD), which could lead to heart failure through a variety of mechanisms, including myocardial infarction and chronic pressure overload. Pathogenetic mechanisms, mainly linked to hyperglycemia and chronic sustained hyperinsulinemia, include changes in metabolic profiles, intracellular signaling pathways, energy production, redox status, increased susceptibility to ischemia, and extracellular matrix remodeling. The close relationship between type 2 DM and CVD has led to the common soil hypothesis, postulating that both conditions share common genetic and environmental factors influencing this association. However, although the common risk factors of both CVD and type 2 DM, such as obesity, insulin resistance, dyslipidemia, inflammation, and thrombophilia, can be identified in the majority of affected patients, less is known about how these factors influence both conditions, so that efforts are still needed for a more comprehensive understanding of this relationship. The genetic, epigenetic, and environmental backgrounds of both type 2 DM and CVD have been more recently studied and updated. However, the underlying pathogenetic mechanisms have seldom been investigated within the broader shared background, but rather studied in the specific context of type 2 DM or CVD, separately. As the precise pathophysiological links between type 2 DM and CVD are not entirely understood and many aspects still require elucidation, an integrated description of the genetic, epigenetic, and environmental influences involved in the concomitant development of both diseases is of paramount importance to shed new light on the interlinks between type 2 DM and CVD. This review addresses the current knowledge of overlapping genetic and epigenetic aspects in type 2 DM and CVD, including microRNAs and long non-coding RNAs, whose abnormal regulation has been implicated in both disease conditions, either etiologically or as cause for their progression. Understanding the links between these disorders may help to drive future research toward an integrated pathophysiological approach and to provide future directions in the field.

## Introduction

Type 2 diabetes mellitus (DM) is a complex metabolic disease in which concomitant insulin resistance and beta-cell impairment lead to hyperglycemia, which is the hallmark of the disease (1). Its prevalence is in rapid and progressive rise, due to the increase in average life expectancy, growing prevalence of obesity, and westernization of lifestyles in developing countries (2, 3), while its long-term complications are the major causes of morbidity, mortality, and exceptional healthcare costs (4, 5).

Cardiovascular disease (CVD) represents a leading health problem worldwide (6). Prospective studies have demonstrated that diabetic patients have a two- to fourfold propensity to develop coronary artery disease (CAD) and myocardial infarction (MI) (7), establishing that type 2 DM is an independent risk factor for stroke and heart disease (8). Indeed, about 70% of type 2 DM at an age ≥65 years die from CVD (7), while type 2 DM patients with no history of CAD have an equal cardiovascular risk as patients with previous MI (9). CVD and type 2 DM share several common pathophysiological features that are summarized in Table 1. Classical cardiovascular risk factors, such as dyslipidemia, hypertension and obesity can also raise the risk of type 2 DM. In particular, insulin resistance and hyperglycemia are associated with a low-grade inflammation, as well as with chronic enhancement of oxidative stress, triggering endothelial dysfunction and promoting atherogenesis (10–12). Among the different soluble mediators associated with the above-mentioned aspects, IL-1β, IL-6, tumor necrosis factor (TNF)-α, and CRP are worth mentioning (13). In addition, it is well documented that type 2 DM is associated with enhancement of platelet and hemostatic activities (14).

**Table 1:** Common pathophysiology of type 2 diabetes mellitus (DM) and cardiovascular disease (CVD).

Status	Description	Reference
Insulin resistance	Insulin resistance is one of the most important antecedent of type 2 DM and CVD	(15)
Inflammation	There is a strong relationship between insulin-resistant states, inflammation, and CVD	(14, 16)
Oxidative stress	Chronic oxidative stress contributes to the pathogenesis of insulin resistance, type 2 DM, and CVD	(17)
Hypercoagulability	Enhanced activation of platelets and coagulation factors is reported in patients with type 2 DM and CVD	(13, 18)
High blood pressure	A positive association exists between hypertension, type 2 DM, and the risk of CVD	(19)
Dyslipidemia	Diabetic dyslipidemia is a major link between DM and the increased cardiovascular risk of diabetic patients	(20, 21)
Obesity	Obesity is a major risk factor for type 2 DM and CVD	(22)

Currently, a number of evidences exists, demonstrating that the interaction of type 2 DM and related cardiovascular risk underpin the progressive nature of the vascular damage, leading to atherosclerosis (23), while it is also proved that lifestyle modifications, such as physical activity and weight loss, counteract CVD risk factors in prediabetic individuals (23, 24). As diabetes shares many risk factors with CVD, while some other ones may be independent, this reinforces the postulate proposed by Stern, according to which both diseases come independently from a “common soil” (20). In this scenario, as type 2 DM and CVD are both complex diseases, common risk factors predisposing to these disorders may include shared genetic factors, a setting that has been only partly elucidated.

Many common single-nucleotide polymorphisms (SNPs) have been already associated with an increased risk of CVD and type 2 DM (25), while their search is still ongoing. In addition, novel links between these disorders come from epigenetic studies. In this review, we will try to address the current knowledge about the genetic links between type 2 DM and CVD, and to evidence their potential pathophysiological role in the context of these diseases. We will dedicate a special focus to the high-mobility group A1 (HMGA1) common variant rs139876191, previously identified by us as a susceptibility locus for type 2 DM (26), and recently also associated with MI (27). In addition, we intend to provide an overview about the epigenetic links between type 2 DM and CVD to widen our understanding about the biological mechanisms that join these disorders. More recently, non-coding RNAs have emerged as key regulators of the pathophysiology underlying both type 2 DM and CVD (28–30), adding up to the fast-growing list of common background in the epigenetic regulation between type 2 DM and CVD. However, these mechanisms are often addressed within a specific pathological context, whereas an integrated approach should be preferred in order to capture all potential interlinks between type 2 DM and CVD.

## Genetic Aspects

### Monogenic Components

Although the most common forms of type 2 DM and the vast majority of CVD are polygenic, Mendelian forms have also been described for both conditions, in which a single gene mutation can trigger the disease (31, 32). In this regard, heterozygous mutations in candidate genes can be at the basis of familial forms of cardiovascular risk factors, including hypertension, hypercholesterolemia and type 2 DM (32). However, such genes do not automatically predispose to both type 2 DM and CVD. For example, recent studies have described a protective role against type 2 DM of *LDL* receptor or *Apo B* gene mutations, the most commonly studied genes for familial hypercholesterolemia. Being this condition characterized by impaired intracellular transport of cholesterol, this suggests a mechanistic role of cholesterol metabolism in type 2 DM (33).

### Genetic Polymorphisms

#### Loci Associated with Type 2 DM and CVD

Many research reports have addressed genetic variants associated with CVD or type 2 DM (34, 35), and the list of loci joint to each specific disease is progressively increasing, mostly due to the power of genome-wide association studies (GWAS), combined with the analysis of large cohorts of patients. Up to now, at least 83 loci have been associated with type 2 DM (36), and more than 30 with CVD (37). As type 2 DM and CVD are linked by common pathophysiological mechanisms, share many risk factors, and display highly correlated phenotypes, different approaches—including candidate gene studies, linkage analyses, and GWAS—have been employed to search for genes predisposing to both diseases. Current findings are summarized in Table 2.

**Table 2:** Genes whose variants are commonly associated with both type 2 diabetes mellitus and cardiovascular disease.

Gene	Relative protein function	Role of genetic variant(s)	Reference
*Adiponectin*	Adipokine with anti-inflammatory and antiatherogenic effects	↑ Risk	(38)

*ADIPOR1*	Adiponectin receptor. Metabolism of fatty acids and glucose	↑ Risk	(39, 40)

*ApoE*	Lipoprotein transport	↑ Risk	(41, 42)

*CDKN2A/2B*	Cyclin-dependent kinase inhibitor. Cell cycle regulation	↑ Risk	(43)

*CELSR2-PSRC1-SORT1*	CELSR2 is part of the cadherin superfamily, involved in contact-mediated communication. Proline- and serine-rich coiled-coil 1 plays an important role in mitosis. Sortilin 1 plays a role in the trafficking of different proteins to either cell surface or subcellular compartments	↓ Risk	(43)

*GLUL*	Enzyme implicated in ammonia and glutamate detoxification, acid–base homeostasis, cell signaling, and cell proliferation	↑ Risk	(44, 45)

*HMGA1*	High-mobility group A1, architectural transcription factor with a role in cell growth, differentiation, and glucose metabolism	↑ Risk	(26, 27)

*HNF1A*	Hepatic nuclear factor 1A, involved in development and metabolic homeostasis	↑ Risk	(43)

*HP*	Haptoglobin. Hemoglobin-binding capacity. Implicated in angiogenesis and in cholesterol-crystallization-promoting activity	↑ Risk	(46, 47)

*Paraoxonase*	Enzyme that protects against lipid oxidation	↑ Risk	(48–51)

*PCSK9*	Proprotein convertase subtilisin/Kexin type 9. Plasma cholesterol metabolism	↓ Risk	(43)

*PHACTR1*	Phosphatase and actin regulator 1. PHACTR1 binds actin and plays a role in the reorganization of the actin cytoskeleton	↑ Risk	(43)

*SOD2*	Superoxide dismutase 2 transforms toxic superoxide into hydrogen peroxide and diatomic oxygen	↑ Risk	(52)

*TCF7L2*	Transcription factor 7-like 2, a member of the Wnt signaling pathway	↑ Risk	(40, 53, 54)

Among candidate genes, several ones involved in pathways pathophysiologically related to both diseases, have been extensively investigated. One of them, *paraoxonase*, synthesizes an enzyme bound to high-density lipoprotein (HDL) particles, with a role in protecting LDL from proatherogenic, oxidative modifications. *Paraoxonase* variants have been described, which lead to reduced enzymatic activity or reduced levels of circulating enzyme, such as the paraoxonase polymorphism Gln-Arg 192, or Met-Leu 54, which are independently associated with both type 2 DM and CVD (48–51). As oxidative stress is a major contributor to atherogenesis in diabetic complications (55), further studies have examined other genes involved in the redox balance. The superoxide dismutase (SOD) 2 is one of the key antioxidant defense systems against free radicals. Ala16Val (rs4880) is the *SOD 2* most commonly described gene variant and resulted in a higher risk to develop CVD in diabetic women (52). Other interesting candidate genes for diabetes and CVD are represented by adiponectin and its pathway. Adiponectin is an adipokine with anti-inflammatory and antiatherogenic effects. Reduced levels of this biomolecule, as in obesity, correlate with increased risk for type 2 DM and CVD, whereas higher levels of adiponectin protect from the risk of CVD in diabetes (56, 57). In patients with type 2 DM, the +276 G/T SNP of the *adiponectin* gene has been reported to be associated with CAD (38). The adiponectin receptor 1 (*ADIPOR1*) gene has been found to be another interesting candidate gene for CVD in diabetic subjects. In particular, common haplotypes tagging three SNPs (rs7539542, rs10920531, and rs4950894) and causing reduced *ADIPOR1* gene expression were found significantly associated with CAD in type 2 DM (39). Furthermore, in type 2 DM, an *ADIPOR1* gene promoter variant (rs266729) has been linked with oxidative stress and cardiovascular risk (40).

One of the most associated spot for MI and CAD, identified by GWA strategies in cohorts of different ethnicities (58, 59), is a 58 Kb non-coding region on chromosome 9p21, localized close to the *CDKN2A* and *CDKN2B* genes, in the context of a known non-coding RNA locus (ANRIL). This same region has turned out to be associated with type 2 DM and several cancers in some studies (60–63). Intriguingly, while the proximity to *CDKN2A* and *CDKN2B*, two genes with a role in cell cycle inhibition and tumor suppression, may explain a causal association with cancer, the 9p21 locus does not contain described genes for CAD, and is not linked with major cardiovascular risk factors, such as plasma lipoproteins, and hypertension. As mentioned before, several studies, but not all, have found the association of this locus with type 2 DM (60–62, 64, 65). In this regard, it has been reported that susceptibility to CAD and diabetes is encoded by distinct, tightly linked SNPs on chromosome 9p21, thereby sustaining an independent association, with the ANRIL locus, of CAD and type 2 DM susceptibility (66). On the other hand, the putative molecular role of this locus in human CVD and type 2 DM has not been yet definitively identified. In fact, while mice lacking the orthologous region on chromosome 4 showed a reduction in cdkn2a and cdkn2b expression in several tissues, as well as increased incidence of cancers and increased proliferation of vascular smooth muscle cells (VSMCs), this condition was not associated with accelerated atherosclerosis (67). Moreover, studies aimed at evaluating CDKN2A/2B and lncANRIL levels in patients have provided conflicting data (68–70), underlying our current limit to interpret results from the non-coding genome. Recently, it has been hypothesized that the regulation of *CDKN2B* gene expression by lncANRIL could be involved in glucose homeostasis (71), while in diabetic patients, high glucose could alter ANRIL expression, favoring cell adhesion and cell proliferation, thereby leading to atherosclerosis (72). Other molecular mechanisms through which lncANRIL are associated with diabetes and its cardiovascular complications, however, remain unclear.

In another important study, 12 loci, previously identified by GWAS as predictors of coronary heart disease (CHD) in the general population, were investigated in three CHD case–control studies of diabetic patients. Among them, five variants, rs4977574 (CDKN2A/2B), rs12526453 (PHACTR1), rs646776 (CELSR2-PSRC1-SORT1), rs2259816 (HNF1A), and rs11206510 (PCSK9), showed a significant association with the risk for CHD also in type 2 DM (43). Among the type 2 DM susceptibility genes investigated by GWAS, the transcription factor 7-like 2 gene *(TCF7L2)* has been identified as one of the most significant (73). *TCF7L2* variants have been found to be associated with CVD in some (40, 53), but not in all (74) reports, although the association between *TCF7L2* risk alleles and CAD was not higher in diabetic individuals. Subsequent studies analyzed the association of three *TCF7L2* variants (rs7903146, rs12255372, and rs11196205) with CAD in 1,650 patients that underwent coronary angiography, and found that these variants were more strongly associated with CAD in diabetic patients than in non-diabetics (54).

Other genetic variants may confer more CHD risk in patients with type 2 DM than in non-diabetic subjects. An example is a polymorphism in the promoter region (−308) of the *TNF-α* gene, whose association with type 2 DM is even stronger in diabetic women (75). Also, as the apolipoprotein E (apo E) polymorphisms are known to modulate the risk for CVD in type 2 DM, many studies, but not all, have shown that the ApoE4 allele is related to a greater susceptibility for CVD in the presence of type 2 DM (41, 42). Another important challenge refers to the identification of diabetes-specific susceptibility genes for CVD. In this regard, interesting studies have addressed the haptoglobin (*HP*) gene polymorphisms. HP is a serum protein that binds free hemoglobin, and prevents hemoglobin-induced oxidation. It is synthesized by two alleles, *HP1* and *HP2*, the former encoded by 5 exons, and the latter by 7 exons, obtained by the intragenic duplications of exons 3 and 4. No significant association was shown between HP phenotype and CVD risk, whereas the *HP2* allele is strongly related to CVD in type 2 DM patients (46). The molecular explanations that may justify this specific association include the reduced ability of HP2, with respect to HP1, to prevent the oxidative stress driven by glycated hemoglobin (46, 76). Further studies have demonstrated that, in a large, type 2 DM-enriched cohort of Americans of European ancestry, the HP2-2 phenotype significantly associates with CVD mortality, triglyceride levels, and subclinical atherosclerosis, in the form of increased carotid-media thickness, but not of calcified arterial plaques (47). Also, a recent GWAS investigated the link between glutamate-ammonia ligase (*GLUL*) gene polymorphism and CHD, demonstrating that the association was specific for type 2 DM patients (44). Further studies confirmed the association of the rs10911021 *GLUL* variant with type 2 DM, and demonstrated that this polymorphism does not affect amino acid metabolism. However, although apparently counterintuitive, it is associated with lower HDL cholesterol levels, and large HDL particles (45).

These and other examples of type 2 DM-specific associated variants, while enriching our knowledge about CVD risk factors, contribute to the debate about the “common soil” hypothesis for type 2 DM and CVD (20, 77). In this context, only few significant loci for type 2 DM and CVD, identified by large-scale GWAS, had shown to be shared between both diseases. Starting from this provocative observation, new strategies have been used to identify novel and ethnic-specific genetic links between CVD and type 2 DM. For example, studies have been carried out using an integrative pathway and network analysis combined with GWAS in more than 15,000 women from three different ethnicities, leading to the identification of eight major pathways shared by type 2 DM and CVD in all ethnic groups (78). In these studies, key driver genes, influencing the extra-cellular matrix composition, such as *COL1A1, COL3A1*, and *ELN*, that had been cross-validated in mouse models for type 2 DM and CVD, have also emerged. Interestingly, few peculiar pathways related to specific ethnic groups were identified (78). In addition, in the past years, attempts have been made to assess a more reliable disease susceptibility for CVD in type 2 DM by analyzing cumulative genetic risk from multiple loci rather than from single SNPs (79, 80). As an example, two genetic risk scores have been successfully used to predict CVD and CVD fatal outcomes using patients from the Diabetes Heart Study (81).

#### HMGA1: An Established Gene for Type 2 DM Risk and a Novel Gene Predisposing to MI

High-mobility group A1 is a small, non-histonic nuclear protein, with pleiotropic effects involved in the regulation of embryogenesis, oncogenesis and tumor progression, cell differentiation, as well as inflammation (82–84). As an architectural transcription factor, it binds to the minor groove of AT-rich regions of DNA, and alters the chromatin conformation, facilitating the assembly and stability of stereospecific DNA–protein complexes called “enhanceosomes,” which drive gene transcription (85–87). Many studies from our group have demonstrated the role of HMGA1 in the transcriptional control of glucose metabolism, being a key regulator of the insulin receptor (*INSR*), insulin-like growth factor binding protein 1 (*IGFBP1*), retinol binding-protein 4 (*RBP4*), *visfatin*, and insulin (*INS*) genes (88–93), as well as an important mediator of insulin action (94). Defects in HMGA1 protein, or the association with functional *HMGA1* variants, among which the most common rs139876191 variant (previously named rs146052672), cause a decrease in *INSR* expression and a trans-ethnic increased susceptibility to either type 2 DM (26, 95–98) or metabolic syndrome (99). Besides its effects on glucose homeostasis, HMGA1 plays a role in adipogenesis and lipid metabolism (100–102), while the HMGA1 rs139876191 variant correlates with body mass index, and reduced HDL levels in patients with metabolic syndrome and type 2 DM (97, 99).

Also, HMGA1 plays a critical role in the development and progression of the atherosclerotic plaque by promoting the proliferation and the migration of VSMCs to the neointima, and by inducing the expression of several inflammatory cytokines, adhesion molecules, including CD44, and chemokines (103, 104). On the other hand, by activating the matrix metalloproteinase 9 (MMP-9), and the vascular endothelial growth factor (VEGF), HMGA1 is essential for vascular repair and neoangiogenesis, whereas its lack causes impairment of both vascular protection from injuries and of neovascularization (92, 105, 106). Recently, the functional HMGA1 rs139876191 variant has been found to be associated with acute MI, independently of type 2 DM or other cardiovascular risk factors, such as hypertension, obesity, and gender, suggesting that *HMGA1* may represent a new candidate gene for acute MI and a marker for cardiovascular risk (27). Although further studies in other populations are needed to confirm this association, due to its pathophysiological role in insulin resistance, glucose homeostasis, lipid metabolism, inflammation and vascular repair, HMGA1 may represent a convincing molecular link between type 2 DM and MI.

## Epigenetic Changes

Epigenetic processes are defined as heritable modifications in gene expression that occur in the absence of changes in the DNA sequence, and include DNA methylation, histone acetylation, and RNA-based mechanisms. These processes are cell-specific, susceptible to modifications, and responsive to the environment, and should be taken into account to better understand otherwise hidden causes of diseases.

### DNA or Histone Modifications

New research investigations have addressed the link between epigenetic factors, type 2 DM and CVD. Hyperglycemia, for example, can induce epigenetic changes that lead to the overexpression of genes implicated in vascular inflammation. In particular, hyperglycemia has been shown to activate the NF-kB signaling pathway in cultured THP-1 monocytes, leading to the production of MCP-1 and other inflammatory factors, and to the expression of adhesion molecules in endothelial cells, providing a plausible molecular mechanism for endothelial dysfunction and atherosclerosis (107). On the other hand, clinical studies have demonstrated that early intensive control of glycemia in diabetic patients is crucial to prevent chronic micro- and macrovascular complications, reinforcing the notion that glycemia may have a longstanding influence on clinical outcomes, a phenomenon called “metabolic memory” (108).

In support of an epigenetic role of hyperglycemia, it has been demonstrated, in aortic endothelial cells, that exposure to high glucose correlates with the inverse acetylation of the histone H3K9/K14 and modified DNA methylation patterns (109). Several histone lysine modifications have also been described following transient high glucose levels that may account for a persistent transcriptional induction of the *RELA* gene, encoding for the p65 subunit of NF-kB, even after subsequent incubation of endothelial cells with normal glucose concentrations (110). Altogether, the net result of this activity leads to the transcriptional activation of some target genes implicated in the endothelial dysfunction, and the repression of other ones (111). Acetylation or hyperacetylation may also occur, being responsible for the increased expression of *HMOX1, MMP10, SLC7A11, MMP1, MCP-1*, and *ICAM* genes (109). Hyperglycemia is, however, not the only inducer of epigenetic modifications. Many other pathophysiologic mechanisms that may be operative in diabetes, independently from glucotoxicity, like ROS, PKC activation, and AGEs have been described to induce also epigenetic changes (112). In particular, ROS production is able to significantly induce the CpG hypomethylation of the p66^Shc^ promoter and, at the same time, an increment in the H3 histone acetylation. Thus, ROS-induced epigenetic modifications are associated with higher levels of p66^Shc^, a mitochondrial adaptor that modulates the intracellular redox state, and with significant activation of PKC, therefore sustaining endothelial dysfunction and vascular damages (111, 112).

Further studies have investigated the associations between epigenetic modifications and cardio-metabolic phenotypes, such as obesity, dyslipidemia, insulin resistance, inflammation, and hypertension, in relation to CVD risk (113). In a recent study, peripheral blood mononuclear cells were used to measure histone deacetylases (HDACs) activity and expression in relation to glycemia, inflammation and insulin resistance in patients with type 2 DM. Low-grade chronic inflammation and insulin resistance induced HDAC3 activity and expression, and correlated positively with circulating levels of TNF-α, IL-6, and other proinflammatory markers, and negatively with Sirt1 expression (114).

Several reports have demonstrated a correlation between DNA methylation and cardiovascular risk. The susceptibility haplotype rs8050136 of the *FTO* gene, a prominent gene associated with increased risk for obesity and CVD, displayed increased levels of methylation (115); a similar mechanism has been hypothesized for the rs9939609 polymorphism (116). In another candidate gene study, an association between IGF2 methylation and lipid profile alterations was found in obese children. In particular, IGF2 hypermethylation was associated with higher triglyceride/HDL-cholesterol ratio, representing an epigenetic marker of metabolic risk (117). Another study that combined genome-wide transcriptome and CpG methylation profiling by array, reported many differentially methylated predicted sites in adipose tissue from insulin-resistant patients compared to controls, which included genes involved in insulin signaling and in the interaction with integrins (118). Altered methylation were also found in *IL18, CD44, CD48, CD38, Cd37, CX3CL1, CXCR1, CXCR2, CXCL1, IGF1R, APOB48R, LEF1, GIPR, GRB10, SIRT2, HDAC4, DNMT3A, LEPR*, and *LEP* genes that were already found to be strongly and independently associated with insulin resistance (118–121). In addition, polarization of adipose tissue macrophages from an anti-inflammatory (M2) to a proinflammatory phenotype (M1) in obese mice was shown to involve the methylation of the PPARγ promoter (122). Finally, there are evidences that MI susceptibility risk may be influenced by epigenetic changes occurring in the prenatal environment (123).

### Abnormalities in MicroRNA (miRNA) Expression

**Figure 1:**
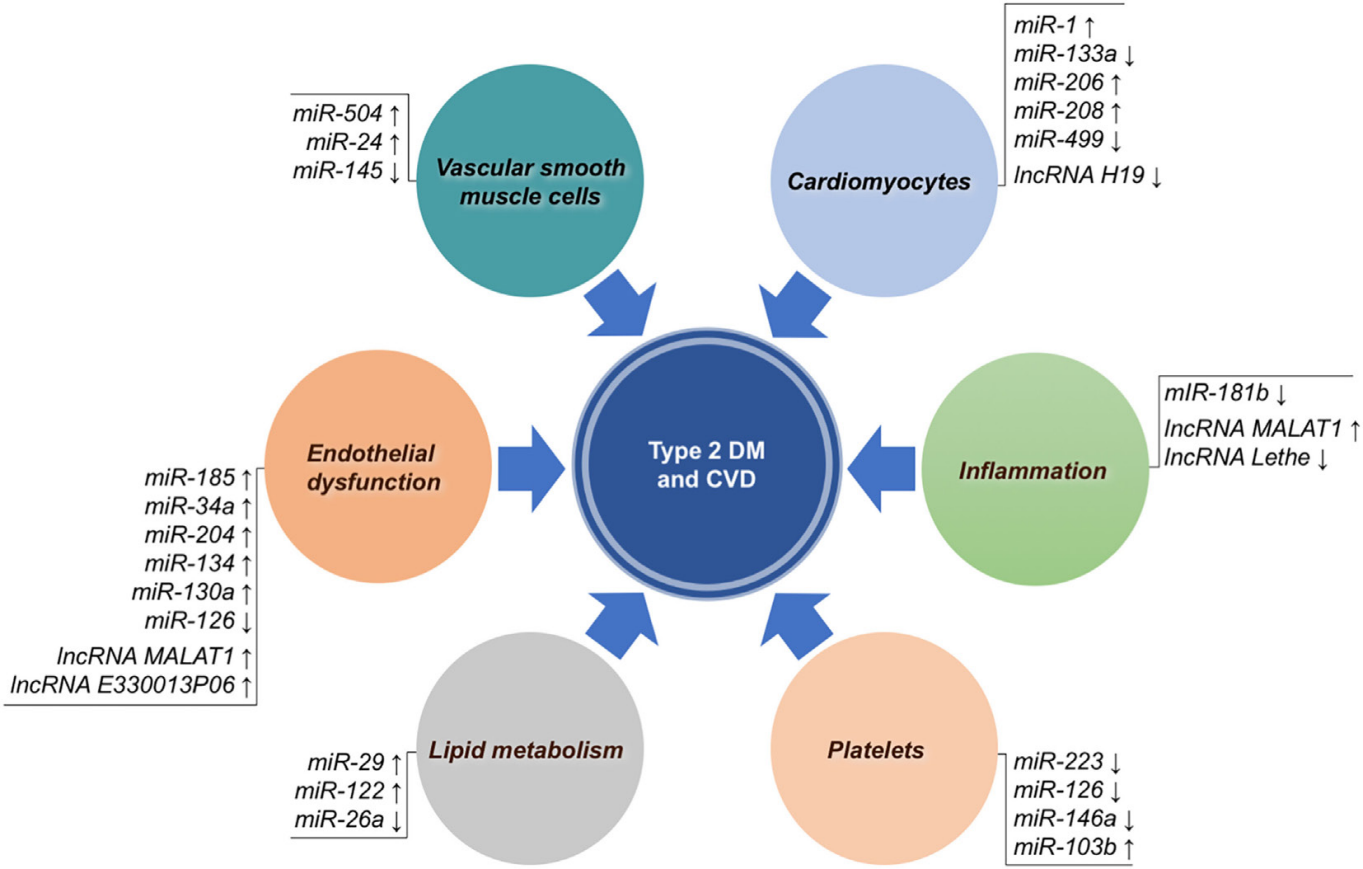
Non-coding RNAs associated with both type 2 diabetes mellitus (DM) and cardiovascular disease (CVD). MicroRNAs (miRNAs) and long non-coding RNAs (lncRNAs) are grouped according to their main biological mechanism involved in atherosclerotic CVD. Arrows indicate overexpression (↑) or underexpression (↓).

MicroRNAs are small single-strand RNA molecules that influence their target genes at a posttranscriptional level, thereby regulating many biological processes. Since their discovery about two decades ago, numerous miRNAs have been described to be associated with a multitude of diseases, including type 2 DM and CVD (28, 124, 125). In particular, with reference to type 2 DM, miRNAs have shown to be involved in regulating beta cell function, insulin response, glucose homeostasis, as well as the pathogenesis of diabetic vascular complications (126, 127). Research in this field has highlighted new mechanistic links between diabetes and CVD (128), with many evidences proving the involvement of distinct miRNAs in the pathological steps that lead to atherosclerosis (Figure 1).

*In vitro* and *in vivo* studies concerning the mechanisms that are responsible for the endothelial dysfunction in diabetes demonstrated that, in the presence of high glucose concentrations, upregulation of miR-185 reduced the expression of the glutathione peroxidase-1 (*GPx-1*) gene, which encodes an enzyme that is important in the prevention of oxidative stress (129); instead upregulation of miR-34a and miR-204 contributed to endothelial cell senescence by impairing SIRT-1 expression and function (130, 131). In the endothelium, miR-126 exerts proangiogenic, and anti-inflammatory activities. At a functional level, it enhances VEGF and fibroblast growth factor activities, contributing to vascular integrity and angiogenesis (132, 133), recruits progenitor cells through the chemokine CXCL12 (134), while it suppresses inflammation by inhibiting TNF-α, ROS, and NADPH oxidase *via* HMGB1 (135). Consistently, miR-126 levels are down-regulated in both myocardial tissue and plasma from type 2 diabetic patients without any known anamnestic data for CVD (136, 137), and in patients with CAD (138), suggesting that it could represent a new diagnostic marker for diabetes and CVD. Other studies in endothelial colony-forming cells, as well as in progenitor endothelial cells (EPCs) exposed to high glucose, demonstrated that miR-134 and miR-130a affected cell motility and apoptosis, respectively (139, 140).

In diabetes, VSMCs loose their contractility and acquire proliferative and migratory properties, facilitating the onset of pathological processes relevant to CVD (141). miR-145 has proved to reduce its level in the presence of high glucose, to impair *myocardin* gene expression *via* Klf4, and to facilitate VSMC proliferation (29, 142). In this context, a role of miR-504 and miR-24 in promoting VSMC proliferation and migration, has also been reported (143, 144).

An important issue is the link between lipid metabolism and miRNAs in diabetic CVD. Several important genes implicated in lipid synthesis or processing, like *FoxA2, Ppargcla, Hmgcs2*, and *Abdhd5* have been shown to be dysregulated by miR-29 in Zucker diabetic fatty rats (145), while HNF-4 alpha was found to be raised by increased levels of miR-122 in diabetic mice and insulin-resistant HepG2 cells (146). Both miR-122 and HNF-4 alpha were able to upregulate the expression of *SREBP-1* and *FAAS* genes, causing abnormal cholesterol homeostasis and high levels of fatty acid and triglyceride synthesis (146). Finally, decreased levels of miR-26a have been reported in obese mice, in which they contribute to increased fatty acid synthesis, and to obesity-related metabolic complications (147).

Platelets are key partaker in CVD and their involvement in the development of cardiovascular complications is strengthened in diabetes (148). Platelets play an important role in the pathophysiology of thrombosis and represent an important source of different RNA species, including pseudogenes, intronic transcripts, non-coding RNAs, and antisense transcripts (149, 150). These molecules can be released by platelets through microvescicles, contributing to the horizontal transfer of molecular signals delivered through the bloodstream to specific sites of action (151). The downregulation of miR-223, miR-126, or 146a observed in diabetic and hyperglycemic patients (137, 152) has been associated with increased platelet reactivity and aggregation (153, 154). In line with these findings, silencing of miR-223 in mice caused a hyperreactive and hyperadhesive platelet phenotype, and was associated with calpain activation through the increased expression of beta1 integrin, kindlin-3, and factor XIII (153, 155). Moreover, the modulation of the expression levels of platelet miRNAs can also be measured in plasma. In fact, plasma levels of miR-223 and miR-126 are decreased in diabetics (137, 156). This leads to the upregulation of the P2Y12 receptor, as well as P-selectin, further contributing to platelet dysfunction (156). As a result of this interaction, activation level of platelets in type 2 DM is increased (149, 156, 157). Consistently with this, circulating miR-223 levels are independent predictors of high on-treatment platelet reactivity (158). Another interesting mechanism linking platelets and diabetes involves miR-103b, a platelet-derived biomarker proposed for the early diagnosis of type 2 DM, and the secreted frizzled-related protein-4 (SFRP4), a potential biomarker of early β cell dysfunction and diabetes. In fact, platelet-derived miR-103b is able to downregulate SFRP4, whose expression levels are significantly increased in pancreatic islets and in the blood of patients with prediabetes or overt diabetes (159). These interesting results identify miR-103b as a novel potential marker of prediabetes and diabetes, and disclose a novel potential therapeutic target in type 2 DM.

Macrophages also play a key role in atherosclerotic plaques. Unbalanced production of proinflammatory molecules from adipose tissue contributes to the polarization of macrophages toward the M1 phenotype and their accumulation within the vessel wall (160, 161). It has been demonstrated *in vitro* and *in vivo* that in the presence of high glucose or in insulin-resistant states, endothelial cells decreased miR-181b expression, while the production of this miR, through the inhibition of AKT Ser 473 phosphorylation, was associated with a M2 anti-inflammatory response, but not with antiproliferative effects (162). These results are compatible with an inhibitory role of miR-181b in atherosclerosis.

Other miRNAs, abundantly expressed in cardiomyocytes, such as miR-1 and miR-133a, seem to be crucial in preventing myocardial dysfunction. Both these miRNAs have been shown to be reduced in ischemic myocardial tissue, in left ventricular hypertrophy, and in diabetic cardiomyopathy (163, 164). Among the molecular mechanisms proposed for miR-133a, the repression of serum response factor, which plays a role in myoblast proliferation, of RhoA (a protein involved in GDP-GTP cycling), Cdc42 (a kinase implicated in hypertrophy), and Nelf-A/WHSC2 nuclear factor (165).

Many cardiac-enriched miRNAs have been reported to be responsive to hyperglycemia, including miR133a, miR-1, and miR-206, with the last two favoring the apoptosis of cardiomyocytes through the negative regulation of the heath shock protein 60 (166). Recent evidences demonstrated that miR-208 and miR-499, together with miR-1 and miR-133, could play a role into the molecular mechanisms leading to the differentiation of stem cells into cardiomyocytes (167). In fact, the involvement of miR-133a in the modulation of contractility was recently demonstrated in streptozotocin-induced diabetic rats (168), in which miR-133a overexpression was able to improve contractility through the upregulation of tyrosine aminotransferase, a known regulator of norepinephrine production and β-adrenergic receptors (168). These latter findings are particularly interesting, as we could recently demonstrate that miR-133a transcoronary concentration has an interesting prognostic potential in patients with CVD (169). Less data is currently available on the involvement of miR-208 in diabetic heart disease. A proposed mechanism for this miRNA implicated a role in the regulation of myosin heavy chain gene expression (170). On the other hand, functional studies showed that miR-499 protects cardiomyocytes from ischemic damage and apoptosis *via* the suppression of calcineurin-mediated dephosphorylation of dynamin-related protein-1 (171).

Specific miRNAs, such as miR-15, -16, -26a, -196a2, and Let-7a (172) are able to modulate HMGA1, whose association with acute MI, type 2 DM, and cardiovascular risk has already been discussed (26, 27, 99). Also, HMGA1 can specifically induce the expression of miR-10b, -21, -125b, -221, -222, or inhibit the production of miR-34a and -603, all of which are involved in several aspects of cardiovascular pathophysiology (173), thereby further supporting the notion that a complex relationship indeed exists between HMGA1 and miRNAs in this context (29, 174).

### Abnormalities in Long Non-Coding RNA (lncRNAs) Expression

Long non-coding RNAs include non-protein coding transcripts longer than 200 nucleotides (175, 176). They have both nuclear and cytoplasmic location and work as signal amplifiers for biological activity, regulating gene expression through a variety of partly explored molecular mechanisms, including the interaction or competition with other RNAs, DNA binding proteins, and specific regulatory DNA sequences (176, 177). New increasing evidences show the involvement of lncRNAs in human diseases (178), such as cardiometabolic diseases (179–182). For example, in the context of atherosclerosis (Figure 1), experimental studies have shown altered expression of lncRNAs in several processes implicated in SMC proliferation, endothelial function, inflammatory cells, lipid metabolism and obesity, as well as with insulin resistance (183), while clinical studies have demonstrated that circulating lncRNAs could be potentially used to predict type 2 DM (182) or the outcome of heart failure (184). However, data from this kind of studies are still initial and in progress. The first lncRNA robustly associated with CVD and type 2 DM has been lncANRIL, a locus identified by GWA studies, already widely discussed in this review in the Section “Genetic Polymorphisms.” Metastasis-associated lung adenocarcinoma transcript 1 (MALAT1) is an lncRNA particularly expressed in the nucleus and physiologically implicated in the regulation of endothelial cell function. It has been demonstrated that hyperglicemia alters MALAT1 expression, leading to micro- and macrovascular damages (185–187). In particular, at a molecular level, MALAT1, by targeting serum amyloid antigen 3, a proinflammatory ligand, has been shown to induce the expression of IL-6 and TNF-α, as well as ROS production, thereby promoting endothelial dysfunction (187). Recently, the lncRNA H19, which has a role in limiting body weight and cell proliferation, was found to be markedly reduced in a mouse model of diabetic cardiomyopathy as a consequence of hyperglycemia (188). In an elegant study, it was demonstrated that lncRNA H19, *via* mIR-675, targets VDAC1, a mitochondrial porin that plays a role in ATP transport, regulating cardiomyocyte apoptosis (188). In other cases, lnc RNAs have been implicated in diabetic vascular complications through mechanisms linked to macrophage-mediated inflammation. By transcriptome profiling of bone marrow-derived macrophages from db/db and diet-induced insulin-resistant type 2 diabetic mice, an increase in lncRNA E330013P06 has been observed, demonstrating that this lncRNA promoted foam cell formation and endothelial dysfunction through the expression of inflammatory genes like *Nos2, IL6* and *ptgs2* (189). Also, a recent study using RAW264.7, as well as bone-derived macrophages, showed that lncRNA Lethe exerted an anti-inflammatory role by inhibiting the translocation of NF-kB transcription factor to the nucleus, and that in the presence of high glucose concentrations, lncRNA Lethe expression was reduced, with a consequent increment in *NOX2* gene expression and ROS production (190).

## Conclusion

In this review, we provide an overlook about the main genetic and epigenetic factors linking type 2 DM and CVD, with a particular emphasis on the pathophysiological mechanisms involved. We addressed known genetic variants shared by both conditions, and the most relevant epigenetic mechanisms involved in their interplay. However, as a lower amount of solid evidence is available to date about epigenetics in this pathophysiological context, further research will be necessary to validate, in patients with type 2 DM, the results obtained so far *in vitro* and *in vivo*, in animal models. A deeper understanding of gene networks, intracellular pathways, and cell-to-cell communication mechanisms will allow the identification of novel biomarkers, as well as new therapeutic targets to exploit in the management of CVD in patients with type 2 DM.

## Author Contributions

SR and BA prepared the first draft of the manuscript; EC was involved in the literature search; AB, CI, and DPF critically revised the manuscript and wrote the final version of the article.

## Conflict of Interest Statement

The authors declare that the research was conducted in the absence of any commercial or financial relationships that could be construed as a potential conflict of interest.
